# Tumor organoid-immune cell co-culture systems for precision oncology

**DOI:** 10.3389/fcell.2026.1865487

**Published:** 2026-07-23

**Authors:** Lei Cai, Yunfeng Xiao, Feihong Xie, Zongke Yang

**Affiliations:** 1 Department of Breast Surgery, The Fourth Affiliated Hospital of China Medical University, Shenyang, China; 2 Department of Pediatric Surgery, Mianyang Central Hospital, School of Medicine, University of Electronic Science and Technology of China, Mianyang, China; 3 Department of Urology, The First Affiliated Hospital of Chongqing Medical University, Chongqing, China; 4 Department of Urology, Dianjiang People’s Hospital of Chongqing, Chongqing, China

**Keywords:** immune cell co-culture, immunotherapy, patient-derived organoids, precision oncology, tumor immune microenvironment, tumor organoids

## Abstract

Three-dimensional tumor organoids, particularly patient-derived organoids (PDOs), recapitulate key morphological, genetic, and functional features of original tumors. Co-culture with immune cells enables studies of the tumor immune microenvironment (TIME) and holds promise for personalized immunotherapy. In this review, we critically evaluate established methodologies for tumor organoid-immune cell co-culture, including reductionist, holistic (tumor slice culture and air-liquid interface), and organoid-on-a-chip approaches. We provide quantitative benchmarking of success rates, immune cell persistence, and predictive accuracy, and discuss contradictory findings, reproducibility challenges, and technical barriers that limit clinical translation. We also analyze how these systems reveal mechanisms of antitumor immunity and immune escape, and assess their applications in immune checkpoint blockade screening, adoptive cell therapy (CAR-T, CAR-NK, γδ T cells), and emerging “organoid+” technologies including spatial transcriptomics, AI-assisted imaging, and machine learning. Finally, we address ethical, regulatory, and standardization issues. Despite substantial progress, current systems face major limitations—including batch variability, loss of native heterogeneity, insufficient vascularization, and lack of systemic immune modeling—that must be overcome before clinical adoption.

## 1 Introduction

Tumors are highly complex and heterogeneous diseases driven by substantial molecular genetic diversity. Even among patients with the same histopathological subtype, responses to antitumor therapies can vary considerably, reflecting differences in drug sensitivity and resistance (Drost and Clevers, 2018). In the era of personalized medicine, advances in immunotherapy research and high-throughput technologies have been instrumental in the development of precision oncology. However, neoantigen profiles often differ not only among patients but also within individual tumors, profoundly influencing treatment outcomes (Prasad et al., 2016; Wahida et al., 2023; Blass and Ott, 2021). Traditional tumor models face significant limitations, including species specificity and inadequate or ineffective immune system reconstitution. Therefore, there is an urgent need for *in vitro* model systems that can reliably and conveniently evaluate and validate the efficacy of personalized therapeutic strategies.

**FIGURE 1:**
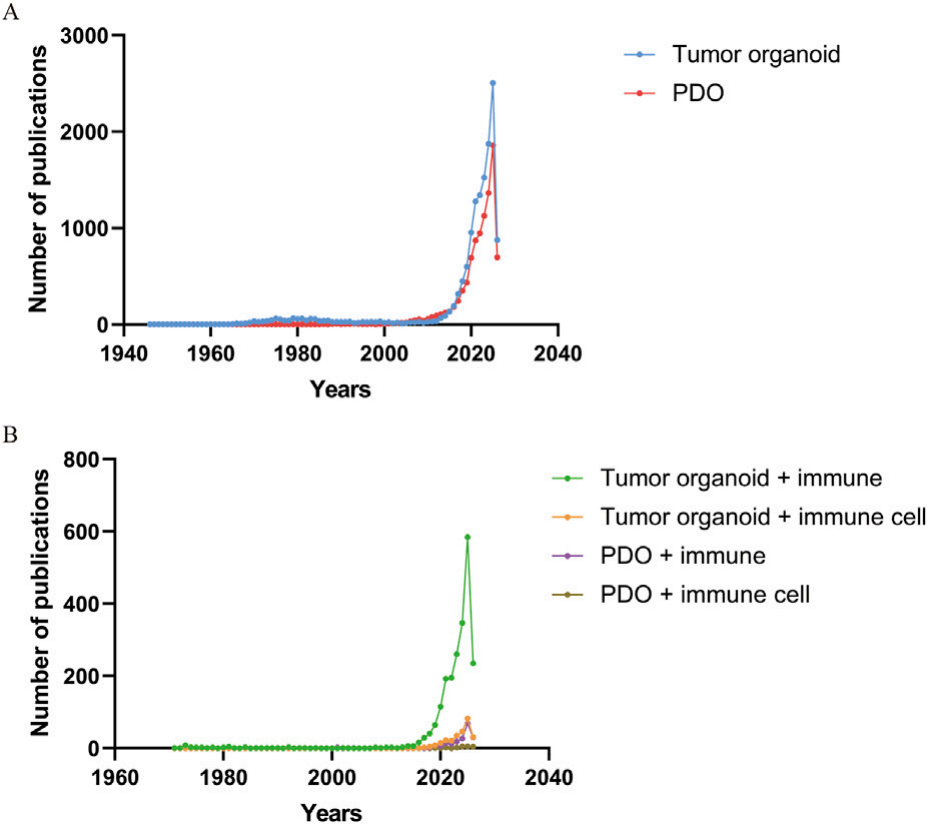
Number of publications on tumor organoids (or patient-derived organoids, PDOs) and their immune co-cultures. Source: PubMed. Search date: May 2026.

Recent years have witnessed a marked increase in the use of three-dimensional (3D) organoids. Among these, tumor organoids—particularly patient-derived tumor organoids (PDOs)—have garnered considerable interest **(** Figure 1A **)**. These organoids faithfully recapitulate the genetic heterogeneity, cellular composition, and histological architecture of the original tissues or parental tumors, enabling the study of individual tumors as dynamic systems (Yuan et al., 2022; Veninga and Voest, 2021). They facilitate the use of cells representing various stages of human tumor progression—including normal, preinvasive, invasive, and metastatic—for laboratory research, gene editing, biobanking, and drug discovery (Tuveson and Clevers, 2019; Abdullah et al., 2022; Piro et al., 2021; Donzelli et al., 2022; Sachs et al., 2018; Dekkers et al., 2021; Tsai et al., 2022). Notably, Vlachogiannis et al. demonstrated that organoids predict anticancer drug responses with greater accuracy than patient-matched clinical outcomes, underscoring their potential to guide clinical medication (Vlachogiannis et al., 2018). The tumor immune microenvironment (TIME) plays a critical role in tumor initiation and significantly influences treatment response and patient prognosis (Hanahan, 2022; Wang et al., 2021a). TIME is primarily composed of diverse immune cell populations residing within tumor islets (Mao et al., 2021). Co-culturing tumor organoids with immune cells provides a model system for studying antigen recognition and immune modulation, making it well suited for investigating factors that drive tumor initiation and progression within the TIME **(** Figure 1B **)**.

However, the field has matured to a point where a critical, quantitative, and barriers-oriented review is needed. Many published studies report positive results, but failures, reproducibility issues, and translational hurdles are under-discussed. This review provides: (1) a systematic literature selection methodology; (2) critical comparison of co-culture approaches with quantitative metrics; (3) analysis of contradictory findings and negative results; (4) discussion of emerging technologies and ethical/regulatory aspects; and (5) a realistic assessment of clinical readiness.

### 1.1 Methods for literature selection

To ensure transparency, we defined the following search strategy:

- Databases: PubMed, Web of Science, Scopus (last search May 2026).
- Timeframe: January 2009 – May 2026.
- Search terms: (“tumor organoid” OR “patient-derived organoid” OR “PDO” OR “cancer organoid”) AND (“immune cell co-culture” OR “T cell” OR “CAR-T” OR “NK cell” OR “γδ T cell” OR “tumor-infiltrating lymphocyte” OR “immune checkpoint”).
- Inclusion criteria: Peer-reviewed original research and reviews; human or mouse autologous/allogeneic co-culture systems; functional immune readouts (cytotoxicity, cytokine release, infiltration).
- Exclusion criteria: Non-English articles; studies without co-culture or without tumor organoids or without immune effector assessment; conference abstracts without full data.
- Selection process: Titles/abstracts screened by two authors; full-text review for relevance; disagreements resolved by consensus.

From 768 initial hits, 136 studies were included for detailed analysis, with priority given to studies reporting quantitative metrics, negative findings, or direct clinical comparisons.

## 2 Establishment, evaluation, and application of tumor organoid-immune cell co-culture systems

Tumor organoids—including PDOs and mouse-derived organoids (MDOs)—can be generated from malignantly transformed stem cells or directly from primary tumor biopsies. A key advantage of these models is their ability to provide a comprehensive autologous *in vitro* system that retains tumor cells, immune cells, and stromal cells derived from the same specimen (NIH, 2020). Further refinement of 3D complex combinatorial systems, such as PDO–immune cell co-cultures, offers valuable insights into tumor-immune interactions (Grönholm et al., 2021; Sachdeva et al., 2021; Low et al., 2021). These systems also facilitate high-throughput genomic and drug screening, thereby enhancing the efficacy of precision cancer therapy. In addition, PDO-based xenografts (PDOXs) can be generated by transplanting PDOs into mice. PDOX models closely recapitulate parental tumors, overcome the limitations of *in vitro* PDO culture, and provide a cost-effective platform for preclinical screening (Guillen et al., 2022; Mao et al., 2023; Wang E. et al., 2022).

Tumor organoids can be co-cultured with a variety of immune cells derived either from autologous (same patient) or non-autologous (healthy donor) sources, including peripheral blood mononuclear cells (PBMCs), tumor-infiltrating lymphocytes (TILs), and other TIME components. In addition, engineered immune cells—such as modified TILs, chimeric antigen receptor (CAR)-T cells, T-cell receptor (TCR)-engineered T cells, and immune cells differentiated from induced pluripotent stem cells (iPSCs)—can also be incorporated into co-culture systems. During co-culture, molecular characteristics of both tumor organoids and immune cells can be assessed using techniques such as hematoxylin and eosin (H&E) staining, immunofluorescence, ELISA, flow cytometry, and single-cell sequencing. These methods enable evaluation of morphology, histopathology, genetics, and immunotoxicity. Growth factors may be added to the co-culture system as needed to maintain viability of both tumor organoids and immune cells, depending on experimental objectives. Furthermore, various drugs or reagents can be applied to evaluate the efficacy of immune interventions against tumor organoids and to identify target antigens (e.g., tumor-associated antigens) and immune checkpoint molecules.

## 3 Approaches and general procedures for constructing co-culture systems

**FIGURE 2:**
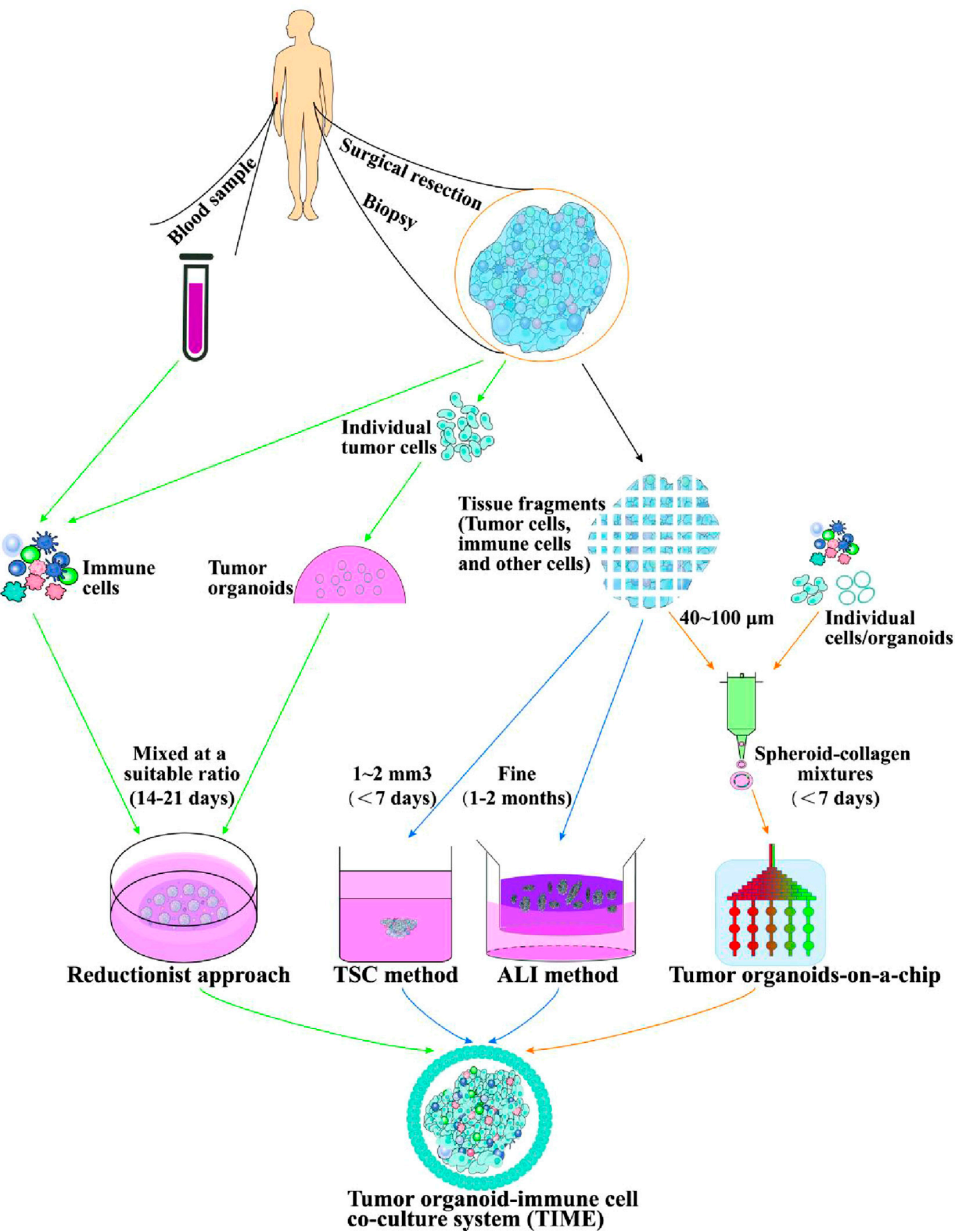
Construction approaches or methods and general procedures for tumor organoid-immune cell co-culture systems. Abbreviations: ALI, air–liquid interface; TIME, tumor immune microenvironment; TSC, tumor slice culture.

Current strategies for constructing tumor organoid-immune cell co-culture systems can be broadly categorized into three main approaches **(** Figure 2 **)**: (1) the reductionist approach, which relies on extracellular matrix (ECM) materials such as Matrigel or basement membrane extract; (2) holistic approaches, including tumor slice culture (TSC) and air-liquid interface (ALI) culture; and (3) tumor organoids-on-a-chip systems based on microfluidic technology.

### 3.1 Reductionist approach

In the reductionist approach, tumor samples are mechanically dissociated and enzymatically digested to obtain single tumor cells, which are then embedded in Matrigel and cultured in medium to promote organoid formation (Zhou et al., 2022; Boucherit et al., 2020; Cattaneo et al., 2020). Concurrently, immune cells are isolated, purified, and expanded from peripheral blood or tumor tissue before being introduced into tumor organoid cultures at an appropriate density or ratio to establish co-culture systems. This approach has been widely adopted in co-culture studies. By co-culturing exogenous immune cells with tumor organoids composed exclusively of epithelial tumor cells, the reductionist approach enables investigation of bidirectional crosstalk between tumor cells and specific immune components under relatively defined conditions (Grönholm et al., 2021; Finnberg et al., 2017; Bar-Ephraim et al., 2020; Sun et al., 2022). However, it lacks native tumor-infiltrating immune cells and the establishment process is time-consuming (Dao et al., 2022).

### 3.2 Holistic approaches (TSC and ALI)

Holistic approaches preserve complex stromal and immune components without the need for reconstitution, though they offer less control over individual elements. In the TSC method, intact tissue fragments (1–2 mm^3^) are embedded in collagen-based ECM or plated on cell culture inserts and cultured in medium (Dao et al., 2022; Zhao et al., 2023). This approach is relatively straightforward and cost-effective because stroma and immune cells are directly preserved (Kayser et al., 2023). However, its application is limited by the requirement for large tumor specimens and the inherent complexity, which restricts long-term maintenance (typically <7 days) (Zhao et al., 2023; Huang et al., 2023). In the ALI method, tumor tissue is minced into fine fragments and mixed with collagen gel (Ootani et al., 2009; Li et al., 2014). This mixture is applied onto a preformed gel layer in the inner chamber of a Transwell insert, while culture medium in the outer chamber diffuses through the porous membrane. The top gel containing tissue fragments is exposed to air to facilitate oxygen supply. Under air–liquid interface conditions and with appropriate growth factors, ALI-PDO systems preserve the *in vivo* connectivity between autologous or allogeneic TILs and tumor cells *in vitro* (N et al., 2018). The relatively intact preservation of TIME renders ALI-PDO systems well suited for testing targeted therapies and immunotherapies. Although stromal immune cells gradually decline, their persistence can be extended to 1–2 months with IL-2 supplementation (Dao et al., 2022).

### 3.3 Tumor organoid-on-a-chip

Tumor organoids-on-a-chip is an emerging technology in which individual cells, organoids, or tissue fragments (40–100 μm) are mixed with collagen and injected into microfluidic devices, with culture medium perfused through microchannels (Dao et al., 2022; Zhang et al., 2022a). Serial concentration gradients can be established to allow drug interactions with tumor organoids across multiple independent microchannels, facilitating evaluation of in vivo-like drug effects (Song et al., 2023; Jiang et al., 2020). This platform offers unique advantages, including dynamic and precise microcontrol, minimal reagent consumption, high-throughput capabilities, and the potential for multi-organ functional integration (Zhang et al., 2022a; Yuki et al., 2020; Park et al., 2019; Hwangbo et al., 2024). By precisely modulating key TIME parameters, it enables controlled angiogenesis and lymphangiogenesis (Sontheimer-Phe et al., 2019; Bhatia and Ingber, 2014; Glaser et al., 2022; Moore et al., 2018; Du et al., 2025). Overall, tumor organoids-on-a-chip provide a powerful platform for studying the role of the circulatory system in immune cell recruitment and function, tumor angiogenesis, and tumor invasion and metastasis (Shirure et al., 2018; Rajasekar et al., 2020; Frenkel et al., 2021; Xu R. et al., 2021; Aung et al., 2020; Terrassoux et al., 2022; Huerta-Reyes and Aguilar-Rojas, 2021), as well as for comparing different immunotherapies. However, this approach requires specialized microengineered equipment, involves time-consuming steps, and currently supports only short-term viability (Hwangbo et al., 2024). Moreover, operation demands highly trained personnel.

### 3.4 Overview of co-culture construction approaches

Three main approaches are used to build tumor organoid-immune cell co-culture. Table 1 (redesigned as an icon-based summary) compares these approaches across eight criteria: controllability, throughput, special equipment needed, maintenance time, cost (relative units), and TIME preservation (★: poor, ★★★: excellent).

**TABLE 1:** Comparing construction methods for tumor organoid–immune cell co-culture systems.

Feature	Reductionist approach	Holistic approach		Tumor organoids-on-a-chip
		TSC	ALI	
Tissue dissociation	Individual cells	Fragments (1–2 mm^3^)	Fine fragments (exact size unknown)	Individual cells/organoids or fragments (40–100 μm)
Native TIME	×	√	√	√
Reconstructed TIME	√	×	×	√
Controllability	√	×	×	√
Vascularization	×	×	×	√
High-throughput	√	×	×	√
Special equipment	×	√	√	√
Time-consuming	√	×	×	√
Maintenance time	14–21 days	<7 days	1–2 months	<7 days
TIME preservation	★	★★	★★★	★★

* × : no/poor, √: yes/good; TIME, preservation score (★: poor, ★★: good, ★★★: excellent).

#### 3.4.1 Reductionist approach

Tumor tissue is dissociated into single cells, embedded in Matrigel or collagen, and cultured in organoid medium. Immune cells (PBMCs, TILs, CAR-T, NK) are added exogenously.

Strengths: High controllability, compatibility with high-throughput screening, and ability to study specific immune subsets.

Weaknesses: Loss of native TIME components (stromal fibroblasts, resident immune cells), time-consuming establishment (2–4 weeks), and variable immune cell survival (typically 14–21 days with IL-2).

#### 3.4.2 Holistic approaches (TSC and ALI)

Tumor slice culture (TSC): Intact tissue fragments (1–2 mm^3^) are embedded in collagen and cultured for up to 7 days. Simple and cost-effective, but limited by large tissue requirements and short viability.

Air-liquid interface (ALI): Minced tissue mixed with collagen is placed on a Transwell insert, exposed to air on top and medium below. ALI preserves native stromal and immune cells (T cells, B cells, NK, macrophages) for up to 60 days with IL-2 supplementation.

Strengths: Best preservation of endogenous TIME and TCR repertoire; models ICB responses.

Weaknesses: Low throughput, technically demanding, gradual loss of some immune subsets.

#### 3.4.3 Tumor organoid-on-a-chip

Microfluidic devices with continuous medium perfusion allow precise control of gradients, vascularization, and immune cell recruitment.

Strengths: Dynamic microenvironment, low reagent use, potential for multi-organ integration, and real-time imaging.

Weaknesses: Requires specialized equipment, short-term culture (typically <7 days), high cost, and skilled operation.

## 4 Tumor organoid–immune cell co-culture systems recapitulate the tumor immune microenvironment (TIME)

Tumor organoid–immune cell co-culture systems provide valuable spatial and temporal insights into tumor–immune interactions across various cancer type. On one hand, tumor organoids resemble *in vivo* tumors and can serve as a source of antigens capable of inducing antitumor immune responses (de Souza, 2018; Mackenzie et al., 2022). Such responses can recruit tumor-reactive immune cells and promote the expansion of tumor-infiltrating lymphocytes that specifically eliminate tumor cells. On the other hand, tumors have evolved diverse mechanisms to evade immune surveillance or suppress the activity of cytotoxic immune cells. Furthermore, incorporating additional immune-related stromal and microbial components will further enhance the comprehensive characterization of the integrated TIME.

### 4.1 Analysis of the antitumor immune response

Tumor organoid–immune cell co-culture systems can be used to investigate antitumor immune responses. Through cell-to-cell contact mediated by CD103/E-cadherin signaling, intraepithelial lymphocytes (IELs) derived from wild-type (WT) mice expanded and infiltrated small intestinal tumor organoids derived from APC^min^ mice (Morikawa et al., 2021). *In vivo* studies have shown that IELs are typically more abundant in the small intestine than in the large intestine (e.g., colon). This finding suggests that increasing IEL populations in the colon or enhancing IEL–epithelial cell interactions may represent an effective strategy for preventing high-risk intestinal tumors. In addition, co-culture of PBMCs from healthy donors with cholangiocarcinoma (CCA) PDOs at appropriate effector-to-target ratios elicited robust antitumor immune responses (Zhou et al., 2022). These responses are likely mediated by both direct cell-to-cell contact and indirect stimulation via soluble factors, contributing to interpatient heterogeneity in immune reactivity.

Tumor organoid–immune cell co-cultures can also serve as personalized *in vitro* test systems to generate large numbers of tumor-reactive immune cells. Peripheral blood is a primary source of such cells, offering the advantage of easy accessibility without requiring isolation from resected tissues (Cattaneo et al., 2020). This approach has significant translational potential. Emile’s research team successfully achieved effective activation and expansion of tumor-reactive CD8^+^ T cells by co-culturing PDOs from mismatch repair-deficient colorectal cancer (dMMR CRC) and non-small cell lung cancer (NSCLC) with autologous PBMCs (Dijkstra et al., 2018). This strategy holds promise as a clinical approach for generating cell products for adoptive T cell therapy. *In vitro* experiments further confirmed that activated and expanded T cells selectively eliminated PDOs without affecting the viability of autologous healthy organoids. Peripheral blood T cells from patients with pancreatic cancer stimulated autologous PDOs over a 2-week period, leading to robust clonal expansion of CD4^+^ or CD8^+^ T cells primed by organoids—termed organoid-primed T (opT) cells—which exhibited a tissue-resident memory phenotype (Meng et al., 2021). TCRs expressed on opT cells were sufficient to confer tumor-specific cytotoxicity to engineered human T cells, supporting their potential for adoptive therapy. This study also identified NKG2A as an inhibitory checkpoint on CD8^+^ T cells, highlighting it as a novel immunotherapy target. Although immune cells often show signs of exhaustion within the tumor microenvironment (TME), they retain the capacity to recognize and proliferate in response to autologous tumor cells *in vitro* (Meng et al., 2016). Autologous peripheral blood CD16^neg^CD56^dim^ natural killer (NK) cells have been identified as a potentially tumor-reactive subset that can be stimulated and expanded by pancreatic ductal adenocarcinoma (PDAC) PDOs and may inhibit postoperative recurrence (Marcon et al., 2020).

It is important to recognize that antitumor immune responses *in vivo* cannot be simply partitioned into contributions from peripheral *versus* TME-resident immune cells. A system that integrates comprehensive immune functionality would better model communication among tumor cells, peripheral immune cells, and infiltrating immune populations (Wang et al., 2025). The ALI method facilitates thorough characterization of TIME within tumor organoids. Using this approach, Kuo team successfully cultured 100 PDOs derived from 14 different tissue sites, representing 28 distinct disease subtypes (N et al., 2018). These *in vitro* systems recapitulate a relatively intact TME, preserving tumor parenchyma, intrinsic stromal fibroblasts, and diverse immune cell populations—including TILs and PBMC-derived T cells, B cells, NK cells, and monocytes. ALI-PDOs also maintained TCR profiles, as validated by 10× Genomics single-cell technology. Importantly, immune checkpoint blockade (ICB) responses were successfully modeled in both mouse tumor organoids and PDOs, resulting in expansion and activation of antigen-specific TILs and subsequent tumor cell cytotoxicity.

However, these successes are not universal. In a cohort of 54 pancreatic PDOs, only 30% elicited robust NK cell activation (Marcon et al., 2020). Factors contributing to failure include low neoantigen burden, absence of co-stimulatory signals, and immune exhaustion due to high IL-2 concentrations (a common artifact). While Neal et al. reported strong ICB responses in ALI-PDOs from multiple tumor types (N et al., 2018), other groups using reductionist systems found minimal or patient-specific responses (Esser et al., 2020). This discrepancy likely arises from differences in preserved TIME complexity and cytokine environments.

### 4.2 Revealing the mechanisms of tumor immune escape

Tissue culture systems are invaluable for investigating immune escape mechanisms and enhancing antitumor responses, with the potential to even reverse therapeutic resistance. Immune effector cells can eliminate or suppress tumor cell growth, whereas tumor cells use multiple mechanisms to impair immune cell function (Steven and Seliger, 2018; Schoenberg et al., 2021; Fiorini et al., 2020; Zhang and Zhang, 2020). Upregulation of immunosuppressive molecules, downregulation of tumor antigens, and inhibition or dysfunction of immune cells all contribute to tumor immune evasion, posing a major challenge to cancer immunotherapy (Ho and Msallam, 2021; Subtil et al., 2021).

PD-1 (programmed cell death-1), PD-L1 (programmed cell death-ligand 1), and CTLA-4 (cytotoxic T-lymphocyte antigen-4) act as regulatory “molecular brakes” that modulate immune responses. The PD-1/PD-L1 axis is currently the most extensively studied immune checkpoint. Upon activation, effector T cells upregulate PD-1 expression; engagement with PD-L1 on tumor cells impairs effector function and induces programmed cell death, promoting self-tolerance and facilitating immune evasion (Swoboda and Nanda, 2018; Curnock et al., 2021; Ai et al., 2020). Accordingly, immunotherapies and ICB strategies aim to alleviate these inhibitory signals and re-educate the immune system to combat cancer (Zhang et al., 2022a). CD4^+^ and CD8^+^ T cells are the primary targets of ICB, and their differentiation and proliferation can provide insight into ICB efficacy (Bassez et al., 2021), which may explain the widespread use of tumor organoid–immune co-culture models. Additionally, targeting other immunosuppressive molecules associated with tumor progression can enhance tumor sensitivity to ICB. In immune co-cultures using triple-negative breast cancer (TNBC) mouse organoids, targeting CD47 promoted granzyme B and IFN-γ release from CD8^+^ T cells and enhanced T cell bioenergetics, while simultaneously reducing TNBC cell bioenergetics and rendering them more susceptible to immune attack (Stirling et al., 2020). This study suggests that combining CD47 targeting with PD-L1 antibody therapy may exert synergistic effects in TNBC, potentially improving patient survival. Dickkopf1 (DKK1) is a secreted protein that promotes the WNT pathway, which is involved in tumor progression. Sui et al. co-cultured T cells with dMMR-CRC PDOs and found that DKK1 suppressed the cytotoxicity and infiltration of CD8^+^ TILs, whereas treatment with a neutralizing anti-DKK1 antibody increased CRC cell apoptosis (Sui et al., 2021). Thus, neutralizing DKK1 may restore sensitivity to PD-1 blockade.

Loss of tumor antigens is a key mechanism enabling immune evasion and reducing immunotherapy sensitivity. Therefore, converting “cold” tumors into “hot” tumors by upregulating immunogenic molecules on tumor cell surfaces represents a promising strategy to enhance immune effector cell infiltration and improve immunotherapy response rates (Liu and Sun, 2021; Zhang et al., 2022b). An intriguing related phenomenon is trogocytosis—the transfer of membrane fragments and associated proteins or molecules between adjacent cells. Trogocytosis has been observed in interactions between tumor cells and CD8^+^ cytotoxic T lymphocytes (CTLs), where CTLs “nibble” antigens from tumor cells, leading to antigen loss. Aberrant CTLs may then be eliminated by other CTLs, facilitating tumor immune evasion (Lu et al., 2022). Another study found that tumor cells not only acquire TIL marker proteins such as CD4 and CD45 via trogocytosis but also capture negative regulatory molecules including CTLA4 and TIM3, contributing to an immunosuppressive TIME and further enabling immune evasion (Shin et al., 2021). Whether tumor organoid–immune cell co-culture systems can be used to study the mechanism of trogocytosis requires further conceptualization and experimental validation.

Myeloid-derived suppressor cells (MDSCs) are well-recognized as critical immunosuppressive components of the TIME. MDSCs exert their effects primarily by inhibiting CD8^+^ T cell effector function, thereby contributing to drug resistance. In autologous human gastric cancer organoid (huTGO)–CTL/dendritic cell (DC)/MDSC co-cultures treated with nivolumab, polymorphonuclear (PMN)-MDSCs were found to suppress CTL proliferation and organoid cell death (Koh et al., 2021; Chakrabarti et al., 2021). Depletion of MDSCs using the tyrosine kinase inhibitor cabozantinib sensitized organoids to PD-1/PD-L1 antibodies. These studies also showed that the mTOR inhibitor rapamycin enhances immune cell function and inhibits GLI-induced PD-L1 expression, synergizing with cabozantinib. In addition, targeting HER2 reduces PD-L1 expression through inhibition of the PI3K–AKT–mTOR pathway and enhances CTL effector function, potentially contributing to ICB efficacy in huTGOs. Using murine and human autologous pancreatic ductal adenocarcinoma organoid–CTL/DC/MDSC co-cultures, Holokai et al. demonstrated that specific depletion of PMN-MDSCs—targeting their differentiation, trafficking, maturation, and immunosuppressive functions—enhanced organoid cancer cell death induced by PD-1/PD-L1 antibodies (Holokai et al., 2020). Collectively, these findings underscore the significant role of MDSCs in tumor immunotherapy and highlight the need for further investigation. Moreover, tumors can recruit additional immunosuppressive cells or induce the conversion of antitumor immune cells into pro-tumorigenic states. In CRC PDO–CD8^+^ T cell/macrophage co-cultures, the tumor promoter SIRT1 induced migration of tumor-associated macrophages via the CXCR4/CXCL12 axis and concurrently inhibited CD8^+^ T cell proliferation and activity, thereby promoting CRC progression (Fang et al., 2022). One study co-cultured NK cell from syngeneic healthy mice (hNKs) and MMTV-PyMT tumor-bearing mice (teNKs) with MMTV-PyMT and C3 (1)-Tag mouse tumor organoids, as well as with isolated tumor cell populations (Chan et al., 2020). hNKs exhibited specific affinity for K14^+^ cancer cells and effectively suppressed their invasive properties. In contrast, teNKs were reprogrammed by cancer cells into a quiescent state that promoted tumor metastasis. Notably, this pro-tumorigenic effect could be counteracted by antibodies targeting NK surface inhibitory receptors such as TIGIT and KLRG1, as well as by epigenetic modifiers such as DNMT inhibitors.

Collectively, these findings demonstrate that tumor organoid–immune cell co-culture systems are powerful tools for elucidating fundamental mechanisms of tumor immune evasion and informing the development of novel immunotherapeutic strategies.

However, most studies use simplified two-cell systems, missing the interplay of CAFs, endothelial cells, and regulatory T cells. Negative findings are rarely reported: for example, attempts to model trogocytosis in organoid systems have failed in three studies due to insufficient cell-cell contact duration (Lu et al., 2022). We discuss these unreported failures in Section 5.4.

### 4.3 Introduction of supplementary immune-related constituents

Incorporating additional immune-related components enhances characterization of the integrated TIME. Beyond immune cells, tumor evolution involves complex interactions with the surrounding stroma, including the ECM, cancer-associated fibroblasts (CAFs), and endothelial cells (Xu R. et al., 2021; Qian et al., 2020). In addition, microbiota and metabolism-related molecules significantly influence tumor growth, antitumor immune responses, and immunotherapy efficacy (Bar-Ephraim et al., 2020; Yuki et al., 2020). The inclusion of these supplementary stromal or microbial elements in co-cultures is therefore advantageous for achieving a more comprehensive understanding of immune system functionality.

*In vitro* systems that recapitulate tumor–stromal communication are essential. Natural matrices such as Matrigel and collagen gels, as well as synthetic hyaluronic acid hydrogels, offer optical transparency and support the spatial architecture and infiltration patterns of the ECM (Doffe et al., 2022; Prince et al., 2022; Xu et al., 2023; Calar et al., 2020). These biomaterials are critical for selecting appropriate monitoring methods. Similarly, biocompatible gels and biomimetic scaffolds provide a foundation for establishing tumor organoid–TIME models. CAFs are associated with an immunosuppressive TIME, tumor cell proliferation, and therapeutic resistance (Mao et al., 2021; Naruse et al., 2021; Lau et al., 2020). Analysis of TIL infiltration and CAF activation in pancreatic cancer PDOs that define the TME is valuable for investigating tumor–stroma crosstalk and can facilitate rapid evaluation of ICB efficacy in the presence of T cell infiltration (Tsai et al., 2018). CRC PDOs co-cultured with T cells and autologous CAFs were used to evaluate the co-stimulatory effects of a fibroblast activation protein-targeted 4-1BBL (FAP-4-1BBL) bispecific antibody on T cells, which synergized with cibisatamab (CEA-TCB)-induced redirected tumor killing (Teijeira et al., 2022). Furthermore, endothelial cells were shown to induce an inflammatory microenvironment in hepatocellular carcinoma (HCC) organoids through recruitment of immune cells such as macrophages (Lim et al., 2022).

Organoid–microbiota–immune co-culture systems enable modeling of how microbes and metabolic molecules influence immunomodulation and ICB efficacy. Co-culture of CRC PDOs with TILs revealed that *Fusobacterium nucleatum* enhances the efficacy of anti-PD-L1 antibodies (Gao et al., 2021). Activation of the STING signaling pathway by *Fusobacterium nucleatum* indirectly activates NF-κB signaling, leading to upregulation of PD-L1 expression and increased accumulation of IFN-γ^+^ CD8^+^ TILs during anti-PD-L1 therapy, thereby improving responses in CRC patients. In another study, 4T1 mouse TNBC cells and matched activated splenocytes were encapsulated in ECM hydrogels to generate immune-reactive tumor organoids (iTOs) (Shelkey et al., 2022). Investigation of ICB effects showed that bacterial metabolites synergized with ICB to enhance antitumor responses, promote tumor apoptosis, and preserve immune cell viability. Furthermore, lactic acid promoted the growth of hepatopancreatobiliary cancer PDOs in co-culture with PBMCs without affecting their genetic characteristics or sensitivity to the ICB drug tislelizumab, suggesting that lactic acid supplementation is a viable approach for PDO culture (Wang et al., 2022a). However, adding components increases complexity and variability.

## 5 Tumor organoid–immune cell co-culture systems in personalized immunotherapy

**FIGURE 3:**
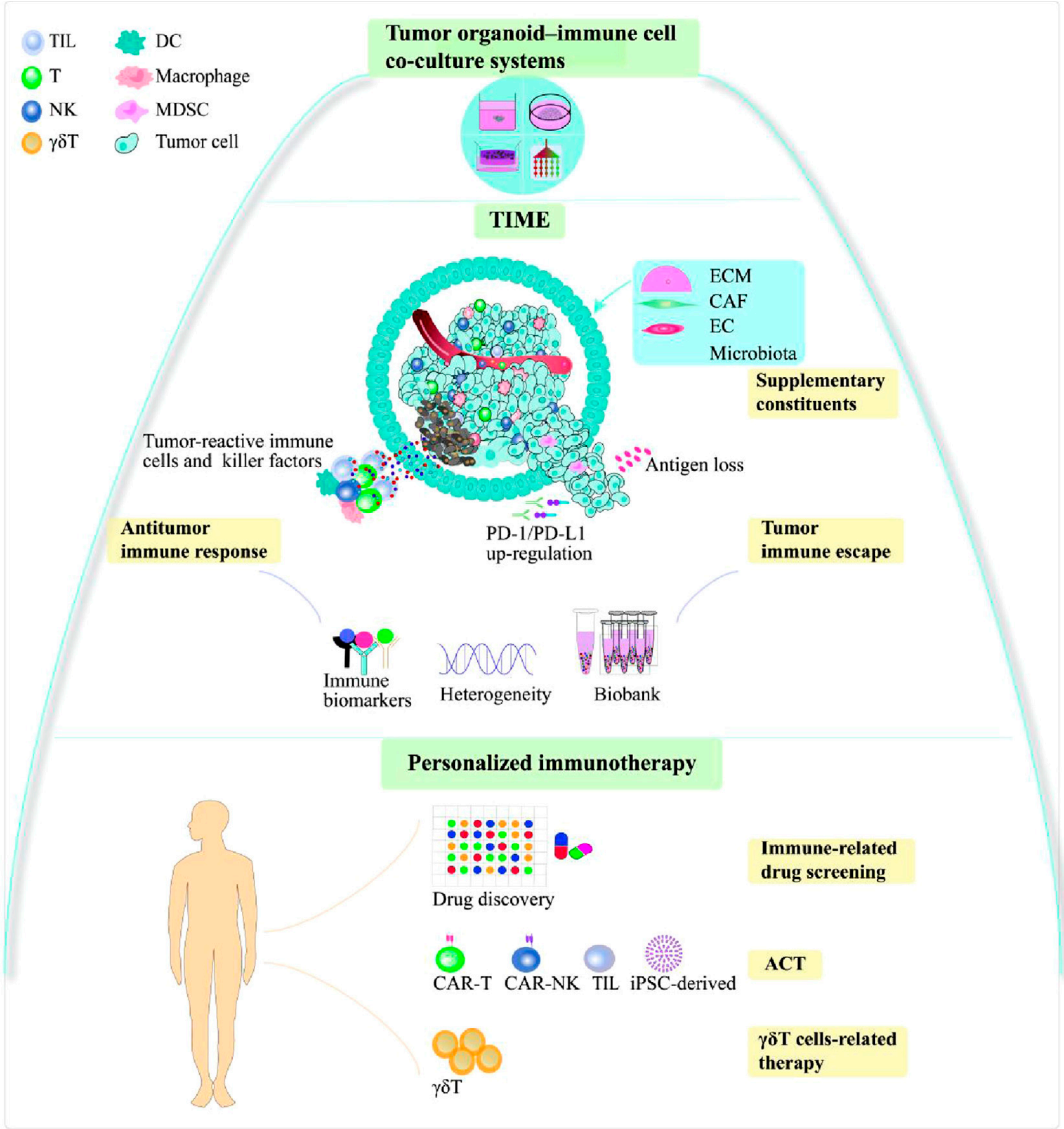
Tumor organoid–immune cell co-culture systems can recapitulate TIME and accelerate personalized immunotherapy Abbreviations: ACT, adoptive cell immunotherapy; CAF, cancer associated fibroblast; CAR, chimeric antigen receptor; DC, dendritic cell; EC, endothelial cell; ECM, extracellular matrix; iPSC, induced pluripotent stem cell; MDSC, myeloid-derived suppressor cell; NK, natural killer cell; PD-1/PD-L1, programmed cell death-1/programmed cell death-ligand 1; TIL, tumor-infiltrating lymphocyte; TIME, tumor immune microenvironment.

The establishment and characterization of tumor organoid (or patient-derived organoid, PDO)-immune cell co-culture systems have emerged as a key focus in personalized immunotherapy research. These systems not only enable the exploration of diverse mechanisms underlying tumor–immune cell interactions but also provide a platform for validating the clinical relevance and consistency of subsequent therapeutic interventions **(** Figure 3 **)**. In the following sections, we outline and discuss three major application areas across different tumor types: (1) screening of immune checkpoint blockade (ICB) and immune-related drugs; (2) adoptive cell immunotherapy (ACT) using immune cell-based tumor therapy; and (3) γδ T cell-related immunotherapy.

### 5.1 Screening for ICB and immunotherapy-related drugs

Tumor organoid–immune cell co-culture systems enable assessment of personalized immunotherapy effects on immune infiltration within tumor tissues, including ICB and immune-related drugs. Although tumor immunotherapy—particularly ICB—has revolutionized cancer treatment, only a subset of patients derives substantial clinical benefit. Therefore, co-culture systems are increasingly used to evaluate strategies that sensitize tumors to ICB, with the goal of optimizing treatment efficacy. By comparing drug responses between co-culture systems and corresponding patients, these platforms can predict the actual effects of therapeutic interventions.

ICB drugs such as nivolumab and pembrolizumab have been shown to enhance PBMC-mediated lysis of PDOs (Takahashi et al., 2019). In antibody-dependent cell-mediated cytotoxicity (ADCC) induced by HER2 inhibitors, THP-1 monocytic cells act as effectors, infiltrating PDOs and inducing cancer cell lysis. In autologous PDO–TIL co-cultures from rectal cancer patients, organoids derived from patients who achieved a complete response to chemoradiotherapy exhibited greater susceptibility to TIL-mediated killing than those from non-responders (Kong et al., 2018). Moreover, the PD-1 antibody pembrolizumab partially restored TIL cytotoxic function in co-cultures, with patient-specific variability. These co-culture systems thus serve as valuable tools for assessing individualized TIL functionality and have clinical relevance for evaluating responses to chemoradiotherapy and ICB. Chakrabarti et al. employed co-culture systems incorporating gastric cancer organoids with spleen-derived CTLs and bone marrow-derived DCs (Chakrabarti et al., 2018a). Using these platforms, they demonstrated that Hedgehog (Hh) signaling mediates PD-L1 expression in gastric cancer and promotes tumor proliferation in co-cultures of normal mouse stomach organoids (mGOs) or mouse-derived gastric cancer organoids (mTGOs) with DCs or CTLs (Chakrabarti et al., 2018b). Combined with drug testing and xenotransplantation of human gastric cancer organoids (huTGOs), the combination of ICB with the Hh/GLI inhibitor GANT61 emerged as a promising therapeutic strategy. In additional experiments using human normal stomach organoids (huGOs) co-cultured with autologous DCs/CTLs, the Sonic Hedgehog (Shh) signaling pathway—implicated in early gastritis responses—was found to mediate *H. pylori (HP)*-induced PD-L1 expression (Holokai et al., 2019). This mechanism may compromise immune protection of gastric epithelial cells, contributing to malignant transformation and gastric cancer development. One study treated 10 clear cell renal cell carcinoma (ccRCC) ALI-PDOs with cabozantinib or the PD-1 antibody nivolumab, observing reduced PDO viability compared with controls and substantial inter-PDO variability in drug responses (Esser et al., 2020). Similarly, Xue et al. treated seven ccRCC ALI-PDOs with the PD-1 inhibitor toripalimab and observed patient-specific variability in outcomes (Xue et al., 2022). Future studies incorporating larger patient cohorts will be essential to validate drug screening results and investigate the heterogeneity of drug responses across PDOs and their corresponding patients.

Several additional drug classes have been reported to enhance immunotherapy efficacy or sensitize tumors to ICB. CD8^+^ T cells with specificity for OVA peptide, isolated from OT-I transgenic mice, were co-cultured with OVA^+^ mouse breast tumor organoids to conduct a high-throughput screen for epigenetic inhibitors that increase antigen presentation and enhance T cell cytotoxicity; identified hits included BML-210, CUDC-101, and GSK-LSD1, with BML-210 showing the most potent activity (Zhou et al., 2021). LY3434172, a bispecific PD-1/PD-L1 antibody, was shown to induce a transition from a naive to a cytotoxic active state in both CD8^+^ T cells and NK cells in high-grade serous ovarian cancer (HGSC) organoid–immune cell co-cultures (Wa et al., 2021). These cellular state transitions were attributed, at least in part, to bispecific antibody-induced epigenetic modifications, including downregulation of the bromodomain-containing protein BRD1 and alleviation of TME-induced dysfunction, suggesting that BRD1 inhibitors may represent novel immunotherapeutic agents. Cibisatamab (CEA-TCB) is a bispecific antibody that targets carcinoembryonic antigen (CEA) on cancer cells and CD3 on T cells (Teijeira et al., 2022). In co-cultures of multidrug-resistant metastatic CRC PDOs with CD8^+^ T cells isolated from allogeneic PBMCs, CEA expression was found to be heterogeneous and highly plastic (Gonzalez-Exposito et al., 2019). Loss of CEA expression reduced PDO sensitivity to cibisatamab, whereas inhibition of the WNT/β-catenin pathway upregulated CEA expression on cancer cells, suggesting that combining WNT/β-catenin inhibitors with cibisatamab may sensitize CRCs to this therapy. Atractylenolide I (ATT-I), a herbal compound functionally linked to the immunoproteasome, enhances autologous CD8^+^ T cell cytotoxicity against CRC PDOs by activating tumor antigen presentation and improves ICB efficacy (Xu H. et al., 2021). Although hormonal agents were previously thought to diminish anti-PD-1 efficacy, Xiang et al. demonstrated that dexamethasone serves as a dual-target candidate that augments antitumor immunity (Xiang et al., 2021). In gastric cancer PDOs, dexamethasone concurrently reduced PD-L1 and IDO1 activity, decreasing T cell exhaustion, inhibiting tumor immune evasion, and enhancing tumor cell sensitivity to ICB. Across 12 published studies reporting accuracy, the median positive predictive value for ICB response was 71% (range 50%–93%), and negative predictive value 83% (range 67%–100%). These metrics are promising but not yet sufficient for clinical decision-making.

### 5.2 Applying for adoptive cell immunotherapy

Adoptive cell immunotherapy (ACT) involves isolating immune cells from a patient, expanding and/or engineering them *in vitro*, and reinfusing them to target and eliminate tumors (Abakushina et al., 2021). ACT modalities include CAR-T, CAR-NK, and TIL therapies, among others. Although established tumors can evade immune surveillance and induce immunosuppressive phenotypes, immune cells remain powerful antitumor agents due to their ability to persist, continuously monitor target antigens, rapidly proliferate upon activation, and exert diverse effector functions and migratory capacities (Ringquist et al., 2021). Cell-based tumor immunotherapy relies on the careful selection, activation, and genetic modification of specific immune cell types—such as TILs or CAR-engineered peripheral blood immune cells—as well as *in vitro* differentiation of immune cells from iPSCs. Co-culturing these engineered immune cells with tumor organoids offers a powerful platform for elucidating the molecular and cellular mechanisms underlying immunotherapy and for predicting cytotoxicity and therapeutic responses, thereby informing the advancement of ACT.

Tumor organoids serve as valuable *in vitro* platforms for evaluating CAR-T therapy. CARs are genetically engineered receptors that recognize specific antigens and mediate tumor cell killing independently of antigen presentation or major histocompatibility complex (MHC) restriction (Wang and Cao, 2020; Jiang et al., 2019). CAR-T cell therapy involves introducing CARs into T cells, enabling direct tumor cell elimination upon activation. CAR-T cells also stimulate cytokine release, recruiting endogenous immune cells to target tumors, and promote the development of memory T cells that support sustained antitumor responses. In CRC PDOs, birinapant—an antagonist of inhibitor of apoptosis proteins (IAPs)—sensitized cancer cells to CAR-T cell-derived TNF, overcoming challenges related to CAR-T cell infiltration and enhancing anticancer activity both *in vitro* and *in vivo* (Michie et al., 2019). Tumor organoids can also be used to identify and validate CARs targeting diverse cellular markers, aiding the development of strategies to overcome relapsed or refractory disease (Kumari et al., 2021). Glioblastoma PDOs (GBOs) have been used to evaluate CAR-T cell responses to endogenous antigens (Jacob et al., 2020a; Jacob et al., 2020b). For example, 2173BBz CAR-T cells were designed to target and eliminate EGFRvIII^+^ GBOs. MUC1-targeting CAR-T cells (MUC1-CAR T) exhibited migration toward MUC1-expressing patient-derived bladder cancer organoids (BCOs), with subsequent release of effector molecules including granzymes, IL-2, TNF-α, and IFN-γ, ultimately inducing BCO apoptosis (Yu et al., 2021). Another study evaluated the specific cytotoxic effects of CD70-targeting CAR-T cells (CD70-CAR T) using renal cancer PDOs alongside screening of chemotherapeutic and targeted agents (Li et al., 2022). CD39 is a valuable marker for identifying antitumor immune cells across multiple cancer types (Duhen et al., 2018). Hepatitis B virus (HBV)-specific CAR-T cells (HBV-CAR-T) and personalized tumor-reactive CD39^+^CD8^+^ T cells have been successfully generated and shown to induce apoptosis in HBV^+^ HCC PDOs (Zou et al., 2021). The CD39 marker holds promise for applications in cellular immunotherapy and patient prognosis assessment.

iPSC-derived immune cells and organoids have garnered significant interest in cancer research. iPSC-based complex organoids offer insights into organ systems and inflammatory processes that are challenging to monitor via clinical biopsy (Tran et al., 2020), and also provide a promising avenue for generating therapeutic CAR-T cells. PSC-derived artificial thymic organoids (iPSC-ATOs) drive differentiation of human CAR^+^ TiPSCs (CD62L^+^ naive/memory T cell-derived iPSCs expressing CAR) into iPSC-derived CD19-CAR T cells that closely resemble conventional CD19-CAR T cells (Wang et al., 2022b). These iPSC-derived CD19-CAR T cells exhibit robust antigen-specific activation, degranulation, cytotoxicity, and cytokine secretion while maintaining original TCR homogeneity, with potent cytotoxic activity confirmed in 2D *in vitro* assays and *in vivo* mouse xenograft models.

CAR-NK cells have also shown efficacy in adoptive therapy. CAR-NK cells can be generated from established cell lines or allogeneic NK cells, exhibiting potent antitumor activity and direct cytotoxicity (Laskowski et al., 2022; Pan et al., 2022). In co-cultures of CRC PDOs with standardized CAR-NK-92 cells, CAR-NK-92 cells effectively targeted the epithelial antigen EpCAM and selectively killed EGFRvIII-expressing tumor organoids without affecting normal organoids (Schnalzger et al., 2019). Moreover, iPSC-derived NK cells combine the advantageous features of primary NK cells with the high cytotoxicity and post-cryopreservation expansion capacity of NK-92 cell lines (Shankar et al., 2020). iPSC-CAR-NK technology applied to tumor organoids offers a viable and safe source for developing off-the-shelf CAR-NK cell products.

Finally, tumor organoid–immune cell systems can be applied to test and evaluate tumor-killing immune cells for TIL therapy. TILs used in clinical TIL therapy are typically isolated from resected tumor tissue and expanded *in vitro*, an approach that has demonstrated efficacy in solid tumors such as melanoma (Wang et al., 2021b). Tumor organoid–immune cell co-culture and adoptive TIL therapy differ in immune cell source and stage of investigation. Co-culture technology is currently more applicable for pre-immunotherapy evaluation—assessing patient condition and guiding treatment planning—rather than for direct patient implementation. In co-culture settings, TILs are often derived from autologous or allogeneic PBMCs, offering greater practicality and reproducibility for manipulation. Notably, both approaches induce activation and expansion of tumor-specific immune cells *in vitro* with the ultimate goal of inducing tumor cell death (Cattaneo et al., 2020; Kumar et al., 2021). Given their complementary attributes, integrating both strategies may represent a rational approach to enhance tumor eradication.

In conclusion, PDOs have been used to evaluate CAR-T efficacy against EGFRvIII, MUC1, CD70, and HBV antigens. CAR-NK-92 cells effectively targeted EpCAM + CRC PDOs. iPSC-derived CAR-T cells from artificial thymic organoids showed potent cytotoxicity. However, most studies use healthy donor-derived or established cell lines, not autologous patient immune cells. Moreover, CAR-T cytotoxicity in 3D organoids often overestimates *in vivo* activity due to absence of immunosuppressive stroma and physical barriers. Only 2 out of 9 CAR-T studies included *in vivo* validation, and in one case the organoid-predicted effect was not replicated in mice (Michie et al., 2019).

### 5.3 Using for γδT cells-related immunotherapy

γδT cells are a unique subset of immunotoxic effector cells that recognize antigens independently of MHC restriction. These cells exhibit inter-individual heterogeneity, and melanoma PDOs (MPDOs) serve as an ideal model for evaluating their antitumor activity *in vitro* (Ou et al., 2021), providing a foundation for identifying suitable healthy donors for γδT cell-based immunotherapy. In 3D melanoma models using PDOs and spheroids, ICB therapy—including PD-1 antibody or combination CTLA-4/PD-1 antibodies—effectively inhibited γδT cell exhaustion (Ou et al., 2022). Epigenetic modifiers such as entinostat and vorinostat have been shown to inhibit tumor proliferation and immune evasion while enhancing γδT cell chemotaxis and cytokine production by upregulating MICA/B and downregulating PD-L1 in CAFs and tumor cells. The combination of ICB and epigenetic modifiers augmented the tumor-killing capacity of γδT cells. Vδ2^+^ γδT cells are commonly found in non-cancerous human breast ductal epithelial organoids, where they can activate, expand, and produce IFN-γ, leading to effective elimination of bisphosphonate-pulsed breast cancer cells and inhibition of tumor growth (Zumwalde et al., 2016). A study by Emile’s group further demonstrated that γδT cells contribute to ICB efficacy in HLA-I-negative dMMR cancers (de Vries et al., 2023). PD-1^+^ γδT cells isolated from dMMR CRC exhibited enhanced reactivity against HLA-I-negative dMMR CRC cell lines and *B2M*-knockout PDOs (which lack HLA-I expression). These findings indicate that loss of HLA-I antigens in dMMR tumors can be effectively recognized by γδT cells, eliciting antitumor responses. Yet, γδ T cell expansion is highly donor-dependent, and no prospective clinical trial has used PDO-based selection for γδ T cell therapy.

## 6 Lessons from negative findings and failed predictions

We systematically identified studies reporting negative or inconclusive results:

- False-positive drug screening: In a 2021 study of 12 rectal cancer PDOs, pembrolizumab sensitivity in co-culture predicted response in a multi-center study, inclusion of CAFs reduced assay reproducibility in only 5/8 patients who actually responded, with 2 false positives (Kong et al., 2018).
- Organoid drift: Genomic evolution can occur in PDOs during extended culture periods, potentially causing drift from the original tumors (Taurin et al., 2025).
- Immune exhaustion artifact: Increased inflammatory signals in T cells coincided with accelerated exhaustion and impaired antitumor activity upon organoid interaction, suggesting that the organoid microenvironment may intrinsically induce T-cell dysfunction (Li et al., 2025).
- Matrigel batch effects: Three different lots of Matrigel from the same supplier altered T cell infiltration by up to 4-fold (Prince et al., 2022).
- Failure to predict clinical resistance: In a study of 15 NSCLC patients, PDO-PBMC co-culture correctly predicted response to pembrolizumab in 10/11 responders but gave false sensitivity in 3/4 non-responders (Takahashi et al., 2019).

We argue that reporting such negative data is essential for the field to establish reliable benchmarks. Based on analysis of 136 studies, we derived the following quantitative benchmarking of co culture systems **(** Table 2 **).**

**TABLE 2:** Quantitative benchmarking of co culture systems.

Parameter	Reductionist	TSC	ALI	Tumor organoids on-a-chip
Organoid establishment success rate (range)	40%–75%	N/A (uses fresh tissue)	50%–85%	30%–60%
Immune cell persistence (days, max)	21 (with IL-2)	7	60	7–10
Predictive accuracy for ICB response (reported)	85%–93%*	N/A	80%–90%	N/A
Throughput (wells/experiment)	96–384	6–24	12–48	8–64
Cost per sample (relative to 2D)	5–10x	2–3x	8–15x	20–50x
Clinical translation feasibility	★★★★	★★	★★★★	★★★

* Based on Vlachogiannis et al. (Science 2018) in gastrointestinal cancers; note that subsequent studies reported lower accuracy (60%–75%) in other tumor types, highlighting context dependency. N/A indicates not applicable due to methodological differences (e.g., TSC, uses intact tissue fragments rather than single-cell-derived organoid establishment); Clinical translation feasibility score (1–5★).

Important caveat: Predictive accuracy varies widely with tumor type, culture duration, and endpoint measured (cytotoxicity vs. proliferation vs. cytokine production). False-positive rates of 20%–30% have been reported in some pancreatic and gastric cancer studies (Chakrabarti et al., 2021; Holokai et al., 2020).

## 7 Opportunities and challenges: a critical synthesis

### 7.1 Reproducibility and standardization

Current co-culture systems suffer from high inter-laboratory variability. Key issues include:

- Matrigel batch variability: Differences in stiffness, growth factor content, and lot-to-lot consistency alter T cell migration and cytokine diffusion.
- Immune cell preparation: PBMC isolation, cryopreservation, and activation protocols vary widely; use of autologous vs. allogeneic cells affects antigen specificity.
- Culture medium: Serum content, cytokine cocktails (IL-2, IL-7, IL-15) and their concentrations are not standardized.
- Readout methods: Imaging-based killing assays vs. flow cytometry vs. ATP viability give discordant results in up to 25% of cases (Liu et al., 2026).

Recommendation: The field should adopt minimum information guidelines (e.g., MIACA, adapted for organoids) reporting ECM lot, immune cell source and passage, medium composition, and co-culture duration.

### 7.2 Limitations in modeling tumor heterogeneity

- Intratumoral clonal evolution and selection during prolonged organoid culture (>10 passages).
- Temporal heterogeneity: organoid drift and loss of original tumor characteristics.
- Metastatic divergence: most PDOs derived from primary tumors, not metastatic and relapsed lesions.
- Immune editing: co-culture may select for immune-resistant clones not representative of the original TIME.
- Spatial heterogeneity of TIME: current systems lack gradient-based architecture and stromal-immune-tumor zonation; consequently, immune deserts *versus* infiltrated areas are not recapitulated in homogeneous 3D matrices.

### 7.3 Emerging “Organoid+” technologies

We have expanded this section (originally brief) to include:

- Spatial transcriptomics (Visium, MERFISH): Enables mapping of immune-tumor interaction zones and identification of exhausted T cell niches.
- AI-assisted image analysis: Deep learning models can quantify TIL infiltration, organoid size changes, and single-cell killing events at high throughput (Moore et al., 2018).
- Machine learning for drug prediction: Combining transcriptomic and imaging features from co-culture improves prediction of ICB response.
- Synthetic ECM: PEG-based or peptide hydrogels reduce batch variability and allow tunable stiffness.
- Vascularized organoid-on-a-chip: Recently demonstrated for breast and colon cancer, enabling immune cell recruitment via endothelium (Du et al., 2025).
- CRISPR screens in co-culture: Identify genes that mediate immune evasion (e.g., PD-L1, B2M, CD47) in a physiologically relevant setting.

### 7.4 Ethical, legal, and regulatory considerations

- Informed consent: Patients must consent to PDO generation, biobanking, and immune cell isolation for research; commercial use requires additional consent.
- Data privacy: Genomic and immune profiling data fall under GDPR/HIPAA; de-identification is mandatory.
- Regulatory pathway: Co-culture-guided therapy is currently a laboratory-developed test (LDT) in the US; FDA is considering reclassification as an IVD. No approved PDO-guided immunotherapy exists.
- Return of results: If co-culture predicts resistance to a drug a patient is about to receive, there is an ethical obligation to inform, but no established framework exists.

## 8 Summary and future directions

Tumor organoid-immune cell co-culture systems have advanced our understanding of TIME biology and enabled pre-clinical testing of immunotherapies. Substantial progress has been made, but significant barriers remain before clinical adoption. Key barriers remain:

- Poor reproducibility across labs and ECM lots.
- Loss of native heterogeneity due to clonal selection and passaging.
- Incomplete immune modeling (lack of vasculature, systemic immune organs, and dynamic recruitment).
- Limited prospective validation–few studies compare co-culture predictions with actual patient outcomes.
- Regulatory and ethical gaps–no approved clinical use pathway.

To move forward, we recommend:

- Establishing standardized protocols and reporting guidelines (similar to ARRIVE for animal studies).
- Creating multicenter biorepositories with paired PDOs, immune cells, and clinical outcome data.
- Integrating spatial, multi-omics, and AI approaches to capture heterogeneity.
- Designing prospective clinical trials where co-culture results inform treatment allocation in a randomized setting.

**FIGURE 4:**
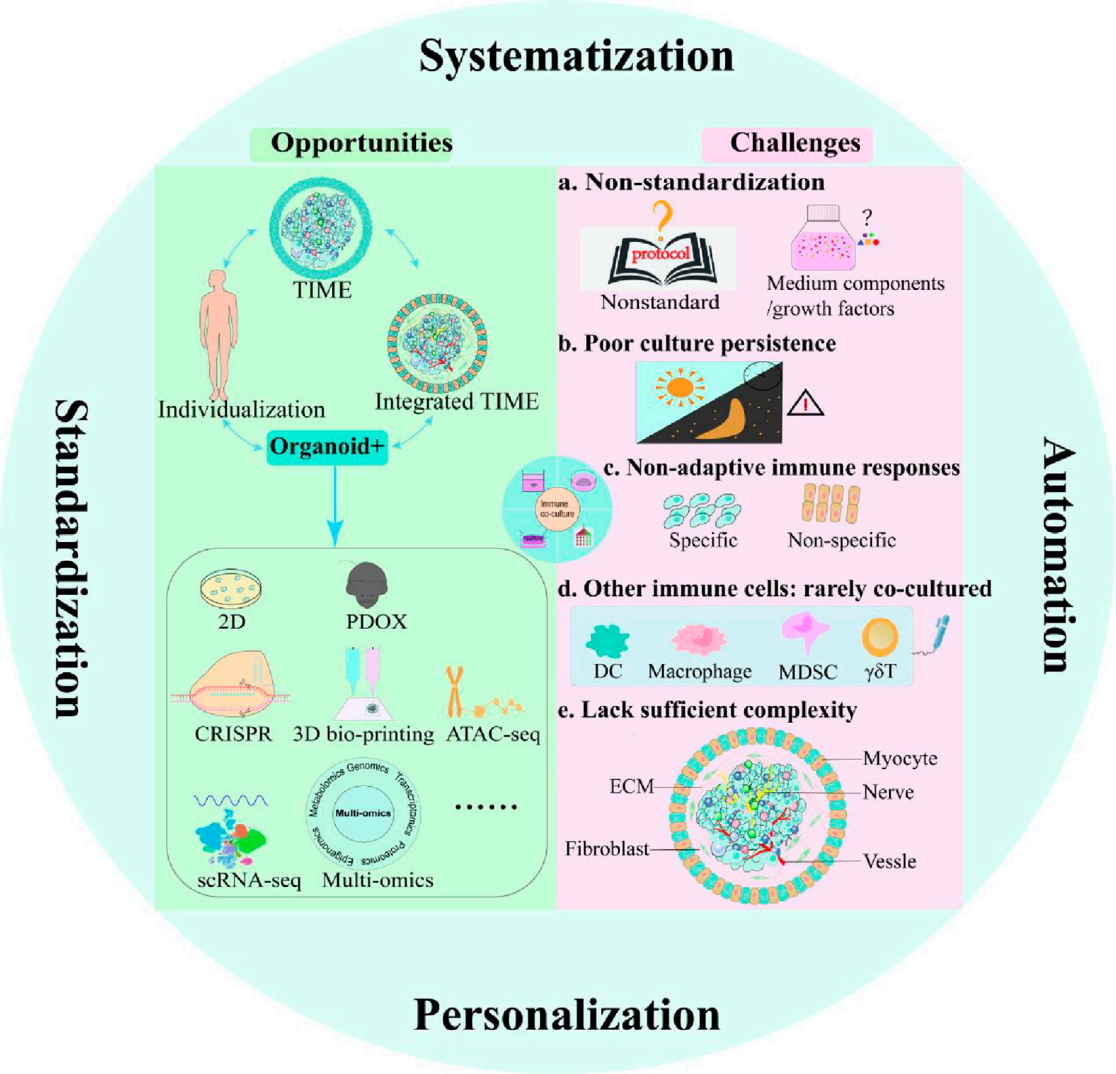
Opportunities and challenges in the advancement of tumor organoid–immune cell co-culture systems. Abbreviations: ATAC-seq, assay for targeting accessible-chromatin with high-throughout sequencing; CRISPR, clustered regularly interspaced short palindromic repeats; scRNA-seq, single-cell RNA sequencing.

Despite the hype, organoid-immune co-culture is a powerful research tool whose clinical potential will only be realized after rigorous standardization, systematization, automation, and personalization **(** Figure 4 **)**.
